# Loneliness and social isolation as predictors of pain medication use transitions in older adults

**DOI:** 10.1038/s41598-026-65304-y

**Published:** 2026-07-31

**Authors:** Alexandre Piot, Daisy Fancourt, Mikaela Bloomberg, Andrew Steptoe, Feifei Bu

**Affiliations:** 1 Clinical Psychopharmacology Unit, Department of Clinical, Educational and Health Psychology, University College London, London, UK; 2 Social Biobehavioural Research Group, Department of Behavioural Science and Health, Institute of Epidemiology and Health Care, University College London, London, UK; 3 Department of Epidemiology and Public Health, Institute of Epidemiology and Health Care, Social Biobehavioural Research Group, University College London, London, UK

**Keywords:** Loneliness, Social isolation, Opioids, Pain medication, Older adults, Health care, Medical research, Risk factors

## Abstract

Older adults are at increased risk of loneliness, social isolation and use of analgesics such as over-the-counter (OTC) medications and opioids. While evidence suggest that loneliness and social isolation may influence patterns of pain medication use, longitudinal studies remain limited. Data from the Health and Retirement Study (2014–2022) were analysed for 9,454 US adults aged 50+. Continuous-time multistate Markov models examined how social isolation and loneliness were associated with the likelihood of transitioning between states of medication use, non-use, and death whilst controlling for sociodemographic and health-related factors. Loneliness was associated with higher risks of initiating both opioids (HR = 1.19, 95%CI: 1.03–1.37) and OTC (HR = 1.12, 95%CI: 1.01–1.25). Conversely, social isolation was not associated with transitions into opioid use, but it was associated with a reduced likelihood of initiating OTC medication (HR = 0.85, 95%CI: 0.77–0.95). Neither factor was associated with medication discontinuation. Loneliness and social isolation are thus differentially related to pain medication transitions in older adults; whereas loneliness may contribute to increased likelihood of initiating medication use, social isolation relates to lower OTC uptake. These findings highlight the need to consider subjective and objective social connection as critical components of safer pain management in older adults.

**Supplementary Information:**

The online version contains supplementary material available at https://doi.org/10.1038/s41598-026-65304-y.

## Introduction

Prescription Opioids (e.g. codeine, morphine, hydrocodone, oxycodone) are a class of drugs primarily used to treat acute pain, but also often used for chronic pain^1^; a common condition in older adults^2^. In 2023, it was estimated that 36% of older adults aged 65 and older had chronic pain in the United States, compared to 24% in the general adult population^3^. The burden of chronic pain likely contributes to higher rates of opioid use among older adults. An estimated 4% to 9% of adults aged 65 and older overuse prescribed opioid medications to manage pain^4,5^. Additionally, high rates of over-the-counter (OTC) pain medication (e.g. ibuprofen, acetaminophen) use and misuse in older adults are a growing concern^6^. Although medications can be essential for pain management, their use, especially opioids, carries considerable potential risks, notably dependence and abuse. Therefore, it is important to understand factors that are associated with pain medication use among older adults.

In the last few decades, there has been a growing awareness of the influence of social connection on health outcomes and management, in particular, loneliness and social isolation^7^. While social isolation refers to the *objective* state of having limited social relationships, roles, group memberships and infrequent social interaction, loneliness refers to the *subjective* and distressing experience of perceived social isolation or a discrepancy between one’s desired and actual social relationships^7^. The estimated prevalence of loneliness in older adults varies depending on the population studied and the measurement tool used, but it is generally estimated at approximately 25% globally^8^. Similarly, a global prevalence of 25% has been reported for social isolation in older adults^9^.

In previous research, loneliness has been linked to increased use of psychotropic medications. Specifically, lonely or socially isolated older adults are more likely to use psychotropic drugs, including analgesics^10^ and exhibit higher odds of polypharmacy, defined as taking 5 or more simultaneously prescribed medications^11–13^. Furthermore, loneliness is associated with higher odds of daily opioids and benzodiazepines use in older adults^14,15^. In addition to direct associations, loneliness and social isolation are also associated with acute and chronic pain in older adults^16^. This suggests that loneliness and social isolation heighten not only the perceived need for pain medication^17^ but also the experience of pain itself, which further drives the use of pain medication^16,18^. Nevertheless, evidence regarding the relationship of loneliness and social isolation with pain medication remains mixed with some studies reporting null findings^12,19^.

Using longitudinal data from the Health and Retirement Study^20^, this work contributes to the understanding of pain medication use in older adulthood by examining how loneliness and social isolation relate to transitions into and out of pain medication use over time. By differentiating between the uptake and cessation of use, this study moves beyond cross-sectional associations to clarify the dynamic role of social connection in pain management, providing novel insights that can inform both clinical practice and public health strategies aimed at reducing medication-related risks among older adults.

## Methods 

### Data

We used data from the Health and Retirement Study (HRS), a nationally representative study of adults aged 50 or above living in the United States^20^. The initial cohort was first interviewed in 1992 and followed up every two years, and the sample is replenished with younger cohorts every six years. Since 2006, HRS has collected psychosocial and lifestyle data in each biennial wave from a rotating random 50% subsample of participants using the Leave-Behind Psychosocial and Lifestyle Questionnaire (LBQ), which included a series of questions on social connection^21^. Since 2016, the main survey included two questions on pain medications, providing a follow-up of 8 years with 4 waves of data (2016–2022). We combined the LBQ subsamples in 2014 and 2016 to form a more complete cohort, which were merged with pain medication data from the main survey between 2016 and 2022. After excluding participants with less than two waves of pain medication data and those with missing data, we had an analytical sample of 9,454 (Figure S1 in the Supplementary Material).

### Measures

Pain medication was measured by two questions: (1) OTC pain medications include such things as Advil, Aleve, Tylenol, aspirin or similar medications. In the past three months have you taken any OTC pain medications for the treatment of pain (yes, no)? (2) Another class of pain medications, called “opioids”, includes such things as Vicodin, OxyContin, codeine, morphine, or similar medications. In the past three months, have you taken any opioid pain medications (yes, no)?

Social isolation was adapted from the Steptoe social isolation index^22^ by assigning one point to each of the following factors: (1) unmarried, (2) less than monthly contact (face-to-face, telephone, or writing/email) with children, (3) less than monthly contact (face-to-face, telephone, or writing/email) with other family, (4) less than monthly contact (face-to-face, telephone, or writing/email) with friends, (5) less than monthly attendance at non-religious meetings or programs of groups, clubs or organisations. The total score ranged from 0 to 5. Participants with a score of 3 or more were defined as being socially isolated. This resulted in a prevalence of 20.9% which was comparable to previous research using the cut-off of 2 in England^22^.

Loneliness was measured using the 3-item revised UCLA loneliness scale. The questions include: (1) how often do you feel lack companionship? (2) how often do you feel isolated from others? (3) how often do you feel left out? Responses to each question were scored on a three-point Likert scale ranging from hardly ever/never, to some of the time, to often. Using the sum score, we had a loneliness scale ranging from 3 to 9, with a higher score indicating a higher level of loneliness. Participants with a score of 6 or more were defined as being lonely^23^.

Covariates included age, sex (female, male), ethnicity (white/Caucasian, black/African American, other), education (no qualification, high school/GED, college, postgraduate), household wealth quartiles. We also accounted for the number of diagnosed physical health conditions, including high blood pressure, diabetes, lung disease, heart disease, stroke, cancer and arthritis. Finally, we included depression as a binary indicator derived from the eight-item Center for Epidemiologic Studies Depression Scale (CES-D), using the cutoff value of 3^24^. To establish temporal orders, all covariates were taken from the previous wave of social connection measures (2012 or 2014), except for age which was defined as age in 2016.

### Statistical analysis

**Fig. 1:**
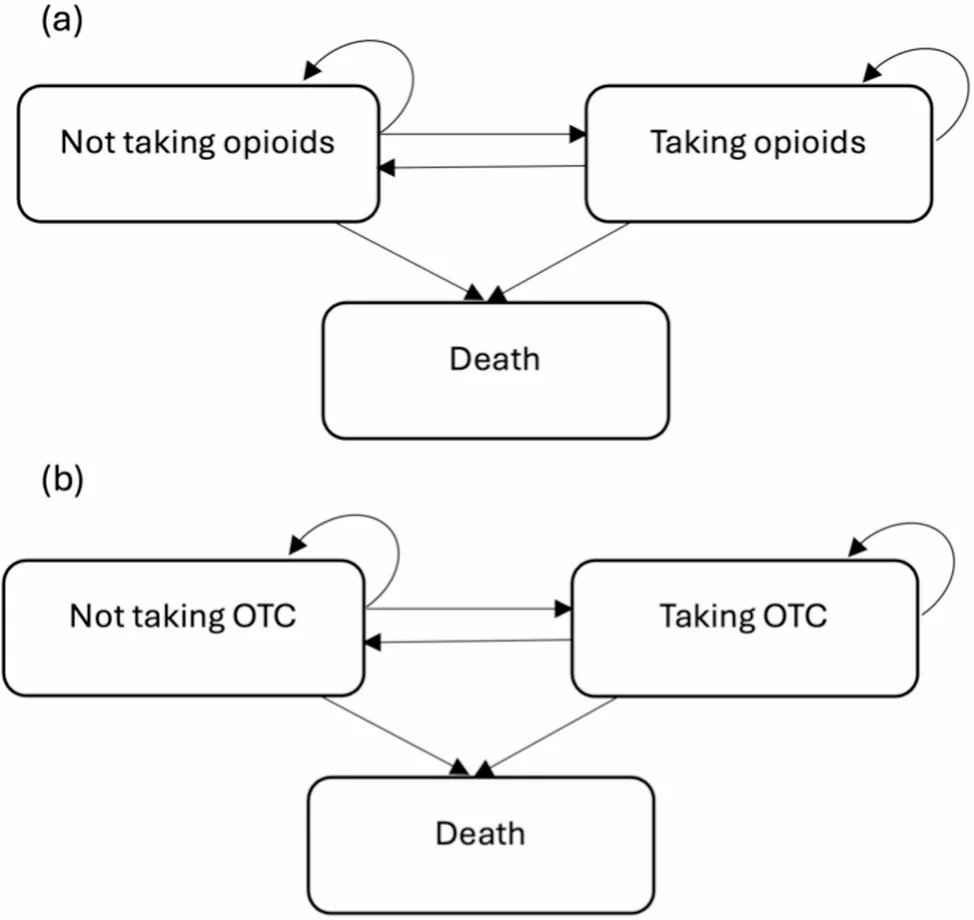
Model specification diagram.

Data were analysed using continuous-time multistate models, separately for opioids and OTC pain medications, and for loneliness and social isolation, respectively. For each set of analysis, we defined three states: (1) not taking opioids/OTC; (2) taking opioids/OTC; (3) death. Transitions were reciprocal between state 1 and 2, but unidirectional between state 1 and 3, and state 2 and 3 (Fig. 1). Death was included as an absorbing state as it precludes the possibility of transitions between medication use states, thereby providing a more accurate representation of the dynamic processes under study. First, we fitted a multistate model with no predictors to obtain predicted state probabilities (Model 0). We then fitted a series of models sequentially: Model 1 (Unadjusted model) included the variable of interest only (loneliness or social isolation), Model 2 (Sociodemographic-adjusted model) included the variable of interest whilst adjusting for sociodemographic covariates (age, sex, ethnicity, education and household wealth), and Model 3 (Fully adjusted model) accounted for all variables in Model 2 and health-related factors (number of health conditions and depression). The covariates were the same for loneliness and social isolation. Data management was performed in Stata V18, but main analyses were conducted in R 4.4.2 using the msm package^25^.

## Results

Of 9,454 participants, 60.6% were females. The mean age at baseline (2016) was 69.83 years (*SD* = 9.58) (Table 1.). Most participants were white/Caucasian (75.1%), followed by Black/African American (17.5%) and those from other ethnic minority backgrounds (7.4%). About a third of participants had a college or postgraduate degree. Over one-fifth of participants (20.9%) were socially isolated and close to one quarter (24.2%) were lonely.

At baseline, 71.5% of participants had taken OTC pain medication, and 14.1% had taken opioids in the last three months (Table 1). During the follow-up period, there were 1,207 transitions from not taking to taking opioids (‘uptake’) and 1,436 transitions from taking to not taking opioids (‘cessation’). Transitions from no opioid use to death occurred 924 times and 182 times from opioids to death. For OTC pain medications, there were 2,751 uptake transitions and 3,113 cessations. Transitions from no OTC use to death occurred 416 times and 690 times from OTC use to death.

**Table 1:** Sample characteristics. Values represent frequencies and percentages for categorical variables and means (standard deviations) for continuous variables.

*N*	Summary statistics
	9,454
**Age**	*M =* 69.825 (*SD =* 9.582)
**N health conditions**	*M* = 1.915 (*SD* = 1.318)
*Sex*	
Female	5,728 (60.6%)
Male	3,726 (39.4%)
*Ethnicity/Race*	
White/Caucasian	7,101 (75.1%)
Black/African American	1,657 (17.5%)
Other	696 (7.4%)
*Highest degree of education*	
None	1,271 (13.4%)
High School/GED	5,069 (53.6%)
College	2,035 (21.5%)
Postgraduate	1,079 (11.4%)
*Household wealth*	
1 (lowest quartile)	2,025 (21.4%)
2	2,210 (23.4%)
3	2,432 (25.7%)
4 (highest quartile)	2,787 (29.5%)
*Taken OTC pain medication in the last 3 months (baseline)*	
Yes	6,760 (71.5%)
No	2,694 (28.5%)
*Taken opioids in the last 3 months (baseline)*	
Yes	1,334 (14.1%)
No	8,120 (85.9%)
*Social Isolation*	
No	7,478 (79.1%)
Yes	1,976 (20.9%)
*Loneliness*	
No	7,170 (75.8%)
Yes	2,284 (24.2%)
*Depression*	
No (CES-D < 3)	7,648 (80.9%)
Yes (CES-D ≥ 3)	1,806 (19.1%)

### Opioid use and loneliness

**Fig. 2:**
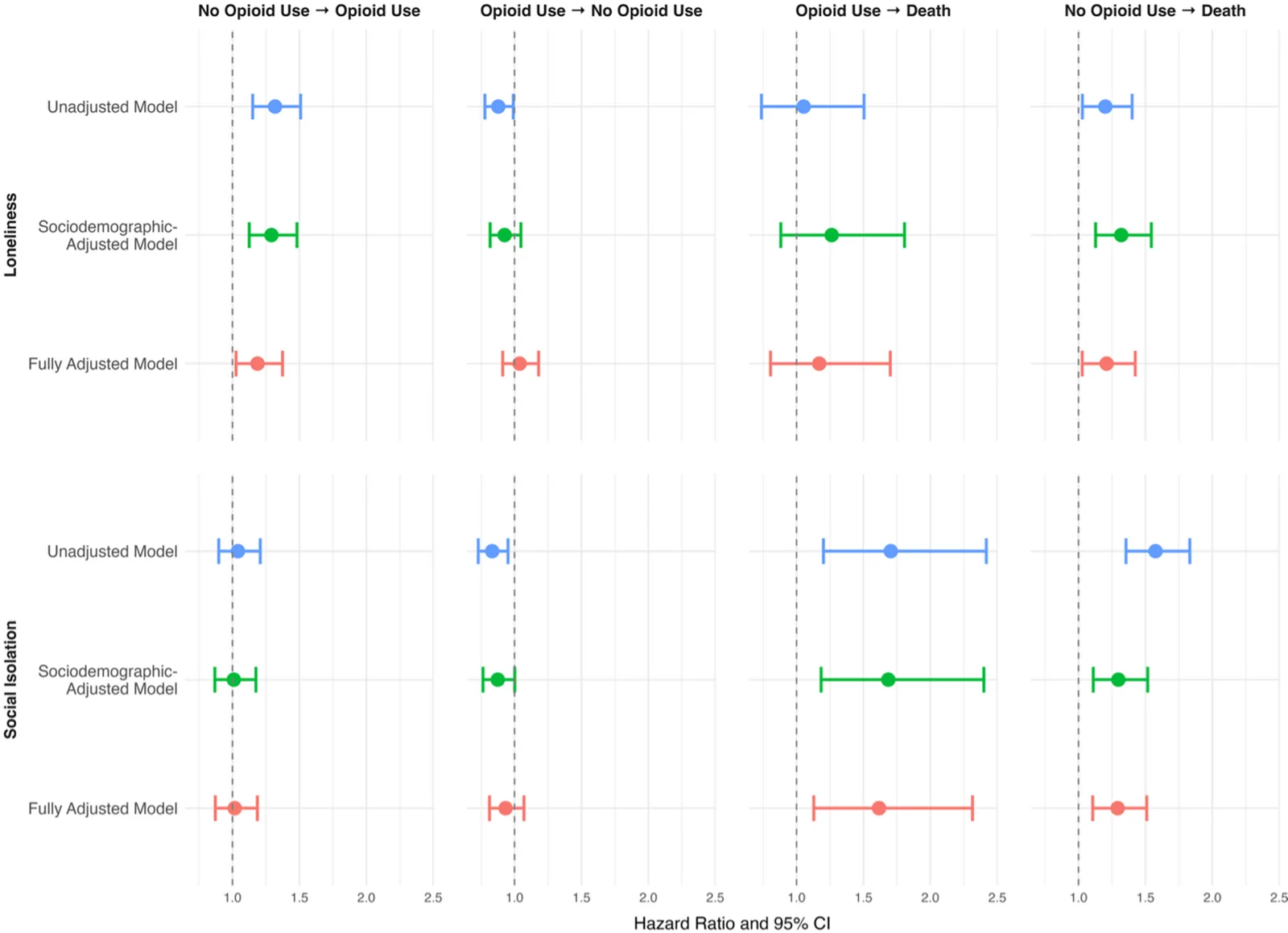
Associations of Loneliness and Social Isolation with Initiation and Cessation of Opioid Use. Hazard Ratios (HRs) and 95% Confidence Intervals (CI) for transitions between different states from the multistate models on opioids pain medication. The unadjusted models include the variable of interest (loneliness or social isolation) only; sociodemographic-adjusted models adjust for age, sex, ethnicity, education, and household wealth. The fully adjusted models account for all sociodemographic variables plus health-related factors, including number of diagnosed health conditions and depression.

In all models, loneliness was associated with a higher risk of uptake transitions from not using to using opioids (Fig. 2a). Even after accounting for sociodemographic and health covariates, loneliness was associated with a 19% higher risk of opioid uptake (Fully adjusted model: HR = 1.19, 95%CI: 1.03–1.37). However, there was no evidence that loneliness was associated with opioid cessation (Fully adjusted model: HR= 1.04, 95%CI: 0.91–1.18).

In addition, loneliness was associated with a higher risk of transitioning from no opioid use to death (Fully adjusted model: HR = 1.21, 95%CI: 1.03–1.42), but no evidence was found for transitioning from opioid use to death (Fully adjusted model: HR = 1.17, 95%CI: 0.81–1.70).

### Opioid use and social isolation

We found no evidence that social isolation was associated with opioid uptake (Fully adjusted model: HR = 1.02, 95%CI: 0.87–1.19) or cessation (Fully adjusted model: HR = 0.93, 95%CI: 0.81–1.07) transitions (Fig. 2b). However, social isolation was consistently associated with a greater risk of transitioning from opioid use to death (Fully adjusted model: HR = 1.62, 95%CI: 1.13–2.32) and from no opioid use to death (Fully adjusted model: HR = 1.29, 95%CI: 1.11–1.51).

### OTC use and loneliness

**Fig. 3:**
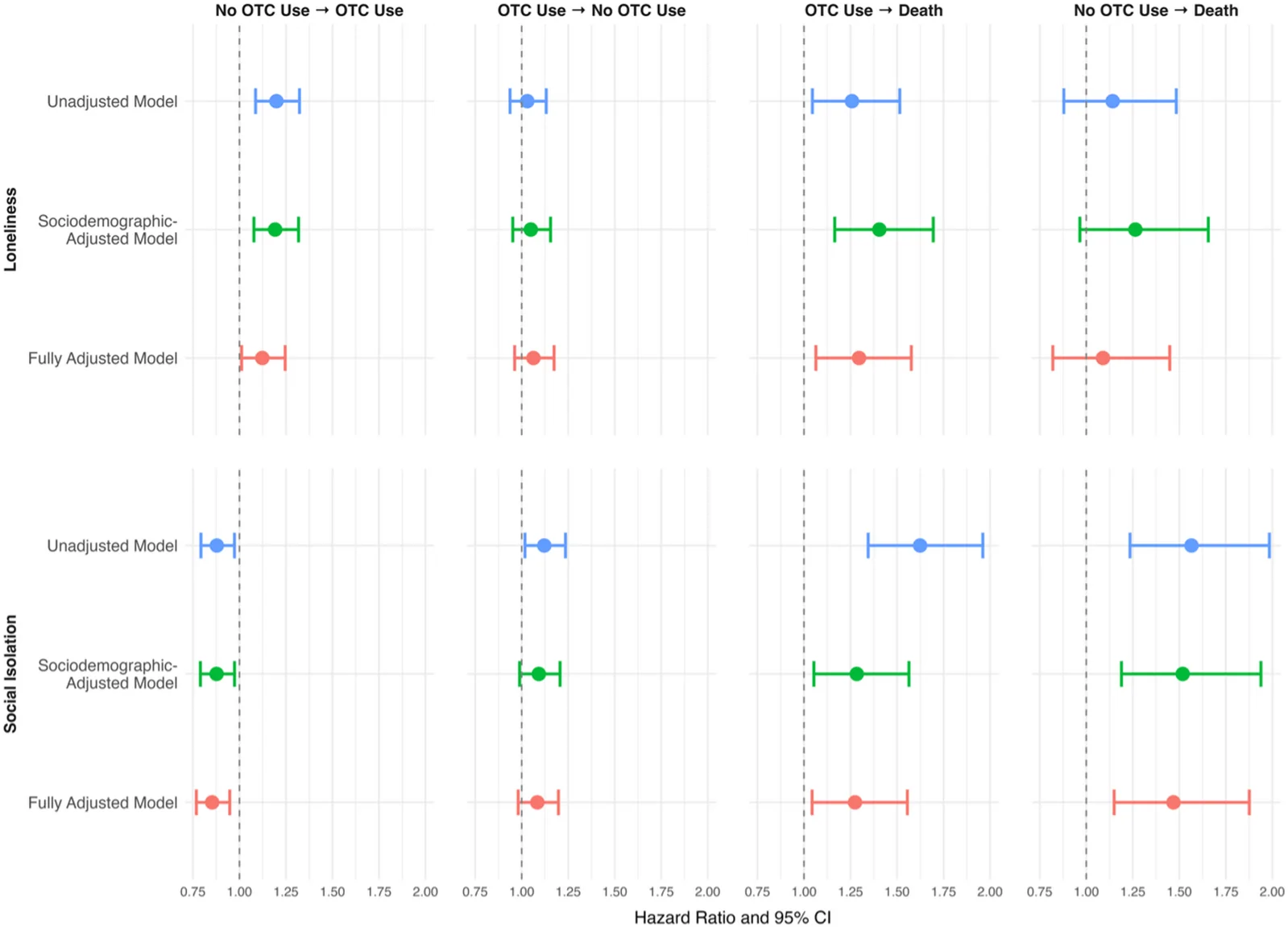
Associations of Loneliness and Social Isolation with Initiation and Cessation of OTC Pain Medication Use. Hazard Ratios (HRs) and 95% Confidence Intervals (CI) for transitions between different states from the multistate models on OTC pain medication. The unadjusted models include the variable of interest (loneliness or social isolation) only; sociodemographic-adjusted models adjust for age, sex, ethnicity, education, and household wealth. Fully adjusted models account for all sociodemographic variables plus health-related factors, including number of diagnosed health conditions and depression.

In the fully adjusted model (Fig. 3a), loneliness was associated with a 12% greater risk of OTC uptake ( 95%CI: 1.01–1.25). However, there was no evidence that loneliness was associated with OTC cessation transitions (Fully adjusted model: HR = 1.06, 95%CI: 0.96–1.17).

Loneliness was associated with greater risks of transitioning from OTC use to death (Fully adjusted model: HR = 1.30, 95%CI: 1.06–1.58), but no evidence was observed for transitions from no OTC use to death (Fully adjusted model: HR = 1.09, 95%CI: 0.82–1.45).

### OTC use and social isolation

As shown in Fig. 3b, social isolation was associated with a lower risk of OTC uptake transitions (Fully adjusted model: HR = 0.85, 95%CI: 0.77–0.95). There was no evidence that social isolation was associated with OTC cessation transitions.

Social isolation was consistently associated with greater risks of transition from no OTC use to death (Fully adjusted model: HR = 1.47, 95%CI: 1.15–1.88) and from OTC use to death (Fully adjusted model: HR = 1.27, 95%CI: 1.04–1.56).

## Discussion

In this study we set out to investigate how loneliness and social isolation were related to older adults’ transition into and out of pain medication use. The multistate modelling approach revealed that for both opioid and OTC pain medications, loneliness was associated with an increased risk of uptake. Social isolation was associated with a lower risk of OTC uptake, but no evidence was found for transitions into opioid use. Both social isolation and loneliness were associated with increased risk of mortality following OTC use, but only social isolation was associated with increased mortality following opioid use. There was no evidence that loneliness or social isolation was associated with pain medication cessation.

Our findings extend a growing body of evidence highlighting how social connection (encompassing both the subjective experience of loneliness and the objective state of social isolation) shape patterns of pain medication use among older adults. In the following sections we discuss how these two dimensions of social connection differ in their implications: while loneliness is more prominently related to the initiation of medication, social isolation is more strongly associated with mortality, reflecting broader structural vulnerabilities.

Regarding the initiation of medication use, our results suggest that the subjective experience of loneliness plays a unique role. Previous work by Vyas et al. ^14^ observed that lonely Canadian older adults had a higher daily use of opioids but not occasional use. Our results extend their work by showing that loneliness was associated with an increased risk of transition into the use of opioids and OTC medications. These associations may reflect a compensatory strategy to address unmet social needs arising from a discrepancy between desired and actual social relationships. Specifically, both opioids and OTC pain medications have been proposed as modulators of social pain, potentially serving as a pharmacological substitute for unmet social connection needs^26,27^. Moreover, loneliness may alter the incidence of pain, or its progression^28,29^, thereby influencing the use of pain medication. Neurobiological theories such as the Brain Opioid Theory of Social Attachment provide mechanistic insight into why social disconnection may lead to increased opioid use. According to this theory, the µ-opioid receptor is a key neurobiological substrate for social bonding, and opioids temporarily alleviate distress caused by social disconnection while reinforcing drug-seeking behaviour^26^. This aligns with our finding that lonely individuals were more likely to initiate opioid use. Furthermore, research investigating experiences of emotional pain preliminarily shows that consumption of acetaminophen led to reductions in reported social pain^30^, though this may be due to overall emotional blunting^31^ and may be a sex dependent effect^32^.

Interestingly, while loneliness was associated with an increased risk of OTC uptake, social isolation was associated with a lower risk of initiating OTC medication. This divergence may reflect distinct underlying mechanisms. While lonely individuals may turn to self-medication to alleviate emotional distress, those who are objectively isolated may lack the social cues and support systems that facilitate access to even non-prescription medications. Although previous cross-sectional analysis has linked social isolation and loneliness to an increased risk of acute and chronic pain^16^, our analyses found no association between social isolation and opioid uptake. We did however observe that loneliness was associated with opioid uptake. Combined these findings highlight that while objectively isolated individuals may experience more pain, their isolation may hinder access to opioids to treat it.

It is of note that neither loneliness nor social isolation was associated with discontinuing opioid or OTC use. This may be due to a compensatory mechanism which underlies the motivation to use pain medication, wherein these medications provide relief from social disconnection, in particular with regards to opioids^33^. Additionally, once medication use has commenced, there may be little motivation to discontinue it, particularly if the user enters a cycle where temporary pharmacological relief is followed by a long-term exacerbation of social isolation and a decreased motivation to seek external social bonds^33^.

We found some evidence linking loneliness to mortality transitions, with divergent patterns between opioid and OTC pain medications. Specifically, for opioids, loneliness was associated with transitions from non-use to death, but not from use to death . For OTC pain medications, however, loneliness was associated with transitions from use to death, but not from non-use to death. These differences could be attributed to the varying pharmacology between the two types of medication and the distinction in subjective and reinforcing effects, the differential severity in conditions they are used to treat, and significant divergence in their accessibility and social acceptability. Nevertheless, social isolation was consistently associated with increased risks of transitioning to death from both use and non-use states for both opioids and OTC pain medication^34,35^. These findings are consistent with the well-established association between social isolation and increased mortality in the literature^22,36^.

To our knowledge, this is the first longitudinal study to examine the relationship between social connection and transitions of pain medication use in older adults using data from a nationally representative cohort study. Distinguishing between opioids and OTC medications, as well as between loneliness and social isolation, provides more targeted insights into the patterns of pain medication and different dimensions of social connection. However, several limitations must be considered. First, because this is a secondary data analysIs, causality cannot be inferred due to residual confounding factors. While several diagnosed health conditions and depression were controlled for, the severity of those conditions, the presence of other unmeasured comorbidities, and medications used could influence both social connection and pain management which may not have been fully captured. We also did not adjust for time-varying pain status or severity, as they are likely either a pathway to or a consequence of medication use. Our estimates represent total associations that include any pain-mediated pathways. Furthermore, the data collected was self-reported and therefore subject to recall bias when reporting pain medication use in the last 3 months. Social desirability bias may have also affected participants willingness to disclose opioid use. Critically, our measures do not capture the context of use such as dosage, frequency, or whether medications were used alongside other substances. We cannot determine if participants were misusing opioids or if the increased risk of mortality observed among OTC users stems from inappropriate use. Since OTC medications are commonly used to treat a wide variety of conditions, it is not possible to determine the specific conditions for which they were used. In addition, the fully adjusted model likely controls for factors on the causal pathway that partly mediate the observed effects. Consequently, the sociodemographic-adjusted model results may be more accurate and less prone to over-adjustment. Nonetheless, it is of note that while the fully adjusted model presents some small attenuations of coefficients, the findings are not materially different, and the conclusions drawn are similar across both models. While death is treated as an adsorbing state in the multi-state model, causes of death are not available, which limits the ability to distinguish deaths directly related to medication from those that were not. This also means that we cannot distinguish between opioid use reflecting the social mechanisms described here and use driven by palliative or end-of-life care, in which opioid prescribing increases substantially as treatment goals shift toward symptom management. Finally, in accordance with open science practices we acknowledge that this study was not preregistered.

The role of loneliness and social isolation in influencing transitions of pain medication use underscores the importance of considering the broader risk environment when considering pain medication use and health practices. As conceptualised by Rhodes^37,38^, drug-related harms are not merely the result of individual behaviours, but rather are shaped by complex interaction between individuals and their social, physical, economic and policy environments. The increased transitions from non-use to opioid use for lonely individuals and opioid use to death for socially isolated individuals illustrate how micro-environment social factors operate within, and are amplified by, macro-environment structural vulnerabilities^38^. Our findings thereby support calls for further consideration of the intersecting risks faced by older adults taking pain medication and implementation of interventions addressing individual level needs (e.g. social prescribing, peer support), and socio-environmental structural reform (e.g. national strategies to address loneliness, enhanced physical and financial accessibility of services and activities).

## Conclusion

This study shows that loneliness and social isolation are each associated with transitions in pain medication use, but in distinct ways. Loneliness was associated with an increased risk of initiating opioid and OTC pain medication use, likely reflecting a compensatory mechanism to alleviate the distress of unmet social needs. In contrast, social isolation was associated with a lower risk of OTC initiation. These patterns support theories of opioid regulation of social homeostasis and broader risk environment frameworks. Our findings not only highlight a significant public health concern but also provide important implications for social wellbeing and effective pain management with ageing populations.

## Supplementary Information

Below is the link to the electronic supplementary material.

Supplementary Material 1

## Data Availability

The datasets analysed during the current study are available in the HRS repository and the RAND Center for the Study of Aging, (https://hrs.isr.umich.edu). Analysis scripts are available at https://github.com/alexandre-piot/HRS-pain-medication-msm.
