# Supplementary material for: Loneliness and social isolation as predictors of pain medication use transitions in older adults
Source: Sci Rep. 2026 Jul 31;16:28353. doi: 10.1038/s41598-026-65304-y (PMC13562570; doi:10.1038/s41598-026-65304-y)
Supplement: Supplementary file 1 [file 41598_2026_65304_MOESM1_ESM.docx]

Supplementary Materials: Loneliness and Social Isolation as Predictors of Pain Medication Use Transitions in Older Adults

Alexandre Piot, Daisy Fancourt, Mikaela Bloomberg, Andrew Steptoe, Feifei Bu

Eligible LBQ 2014/2016

(n=13297)

Main survey 2016-2022

(n=24768)

Merged

(n=12649)

≥2 waves of data

(n=11078)

< 2 waves of data

(2016-2022)

(n=1395, 11.2%)

Not merged with main survey (n=648, 4.9%)

Not merged with LBQ

(n=12119, 48.9%)

Analytical sample

(n=9454)

Missing data

(n=1624, 14.7%)

*Figure S1*. Sample selection diagram. Data were obtained through from the Health and Retirement Study (available at: <https://hrs.isr.umich.edu/>).
